# Body mass index and self-rated health in East Asian countries: Comparison among South Korea, China, Japan, and Taiwan

**DOI:** 10.1371/journal.pone.0183881

**Published:** 2017-08-28

**Authors:** Jin-Won Noh, Jinseok Kim, Youngmi Yang, Jumin Park, Jooyoung Cheon, Young Dae Kwon

**Affiliations:** 1 Department of Healthcare Management, Eulji University, Seongnam, Korea; 2 Global Health Unit, Department of Health Sciences, University Medical Centre Groningen, University of Groningen, Groningen, the Netherlands; 3 Department of Social Welfare, Seoul Women's University, Seoul, Korea; 4 National Institutes of Health Clinical Center, Bethesda, Maryland, United States of America; 5 Department of Nursing Science, Sungshin University, Seoul, Korea; 6 Department of Humanities and Social Medicine, College of Medicine and Catholic Institute for Healthcare Management, The Catholic University of Korea, Seoul, Korea; Medical University of Vienna, AUSTRIA

## Abstract

There have been conflicting findings regarding the relationship between body mass index (BMI) and self-rated health (SRH) worldwide. The purpose of this study was to examine the association between BMI and SRH by comparing its relationship in four East Asian countries: South Korea, China, Japan, and Taiwan. Using data from the East Asian Social Survey, the relationship between weight status and SRH status was investigated and compared between four countries, China, Japan, South Korea, and Taiwan. An ordinal logit regression model was estimated for each country, and the results were compared. We found that the relationship between weight status and SRH status differed across the four countries. In China, people who were overweight reported better SRH scores than those of normal weight, whereas in Japan, obese and severely obese people reported poor scores. In contrast, South Koreans who were underweight, obese, or severely obese reported poor ratings of health status than those of normal weight. In Taiwan, however, no differences in respondents’ weight status were found across SRH scores. There were notable differences in the relationship between BMI and SRH status in four East Asian countries. Individual countries should consider these relationships when designing and implementing obesity intervention programs.

## Introduction

Obesity is a major global health concern, with a prevalence of more than 1.9 million adults worldwide in 2014. The prevalence has grown steadily and has nearly doubled since 1980 [1]. Obesity and overweight can result in increasing risk of non-communicable illnesses such as heart disease, stroke, type 2 diabetes, and certain types of cancer [2]. In this regard, obesity can increase the burden of disease with immense economic costs [1].

Self-rated health (SRH) (also known as self-assessed health or self-perceived health) has been defined as “an individual’s or group’s perceived physical and mental health over time” [3,4]. This concept develops subjectively from many factors such as health behaviors, general physical function, and specific health situation [5,6]. SRH has been described as a powerful independent predictor of morbidity and mortality, in addition to specific biomedical health indicators [3,5]. Furthermore, it is a relatively stable construct in adults, which is consistently related to physical health status [3].

A growing body of literature has investigated the relationship between underweight and overweight/obesity, calculated as body mass index (BMI), and SRH. The results, however, are controversial; some studies reported that underweight and/or overweight/obese status were associated with poorer SRH [5–9], whereas other studies did not observe any association [10]. In addition, the major component of reporting the relationship between BMI and SRH relies on the context of the individual’s socioeconomic and cultural background [11–14]. A cross-country study among diverse racial/ethnic Americans reported that the association between BMI and SRH significantly differed by race/ethnicity; Whites and Hispanics had poorer SRH as BMI increased while Blacks and Asians showed better SRH as BMI increased [14]. Another cross-country study found the relationship between BMI and SRH differed by gender in low-income countries compared to middle-income countries [9]. These results suggest that perceptions and experiences of body weight and health found across different cultural groups affect what is reported and how it is illustrated [9,14]. For example, in developing countries, overweight and obesity might be considered a sign of wealth or health. As obesity rates increase, however, the stigma of excess weight and the promotion of slim ideal body types are growing globally [15]. In some developed countries, people perceive being overweight or obese as a disease rather than a risk factor for chronic diseases [9].

Given different socioeconomic and cultural factors across countries, locally designed health promotion programs should be considered specific to each country rather than universal programs [11,14]. A cross-national comparative study using nationally representative data could help clarify the relationship between BMI and SRH by region, providing insight into tailored health promotion programs for each country. The majority of studies, however, have sampled Western populations, and empirical evidence is lacking in Asian populations. The purpose of this study was to investigate the relationship between BMI and SRH by comparing four neighboring East Asian countries—South Korea, China, Japan, and Taiwan—that are at different stages of socioeconomic development and have different cultural backgrounds.

## Methods

### Data and subjects

Data from the East Asian Social Survey (EASS) were utilized in this study. EASS data are accessible via a public database (http://www.eassda.org) without any restriction. The EASS was made available by collaborative efforts among China, Japan, South Korea, and Taiwan. The EASS Data Archive is housed at Sungkyunkwan University in Seoul, Korea, which is responsible for the management and distribution of data to potential data users. The EASS has been conducted biannually since 2006 and, specifically, this study analyzed the 2010 module of the EASS because the 2010 module included health-related questions. The sample population of Japan were men and women, age 20–89 years, and that for Korea, Taiwan, and China were men and women 18 years or older. Each of the participating countries utilized multi-stage stratified random sampling for their sampling methods and response rates ranged from 49.7% (Taiwan) to 72.0% (China). In the 2010 module of EASS data, there were 3,866 participants from China, 2,496 from Japan, and 1,576 from South Korea. Out of 2,199 participants from Taiwan, we excluded a sub-group of 1,064 who were not asked for subjective health condition question, which resulted in 1,135 valid cases included in further analysis. There were 198 respondents who did not provide BMI-related data, and these respondents were excluded from the analysis.

Our study has been performed in accordance with ethical standards. This study was also approved by the Institutional Review Board of Seoul Women’s University (SWU IRB-2017-6) with a waiver for informed consent because the data obtained from an already public database and analyzed anonymously.

### Variables and measurement

SRH status was measured by asking “In general, would you say your health is…?” using a five-point Likert scale (1 = poor; 5 = excellent). BMI was defined as weight in kilograms divided by height in meters squared, and was calculated using the respondents’ self-reported weight and height. Using BMI scores, this study further classified the participants as underweight (under 18.5 kg/m^2^), normal weight (between 18.5 kg/m^2^ and 23.0 kg/m^2^), overweight (between 23.0 kg/m^2^ and 25.0 kg/m^2^), obese (between 25.0 kg/m^2^ and 30.0 kg/m^2^), or severely obese (over 30.0 kg/m^2^) based on the World Health Organization’s classifications suggested and revised for the Asia-Pacific region. Other covariates such as age in years, gender (0 = male; 1 = female), marital status (1 = married or cohabiting; 0 = otherwise), region (1 = urban; 0 = otherwise), education in years, and other health-related behaviors such as smoking (1 = never smoke; 0 = otherwise) and alcohol consumption (1 = never drink; 0 = otherwise) were also included in the analysis.

### Statistical analysis

A set of descriptive analyses stratified by country were conducted to summarize the characteristics of the samples. Because SRH status, the outcome variable of this analysis, was measured using a five-point Likert scaled question, the relationships between SRH status and weight status were analyzed using a set of ordinal logit regression models, one for each country. Stata MP 14 (StataCorp, College Station, TX, USA) was used for data management and analyses, and the threshold for the significance test was *p*<0.05 (two-sided).

## Results

Characteristics of participants from the four Asian countries are summarized in Table 1. The proportion of people with normal weight was lowest in Taiwan (40.99%) and highest in Japan (51.07%) and proportions of obese and severely obese were lowest in Japan (16.57% and 2.84%, respectively) and highest in Taiwan (27.97% and 4.74%, respectively). Those who reported ‘poor’ health status were most prevalent in Taiwan (14.45%) and least prevalent in Japan (3.93%). These differences in terms of weight status [Chi-squared (12) = 121.22, *p*<0.001] and SRH status [Chi-squared (12) = 1,811.97, *p*<0.001] were significant. There was no difference among the four countries for gender distribution of the study participants [Chi-squared (3) = 3.67, *p* = 0.300]. However, there were significant differences in terms of marital status [Chi-squared (3) = 253.77, *p*<0.001], living in an urban area [Chi-squared (3) = 419.11, *p*<0.001], smoking status [Chi-squared (3) = 95.38, *p*<0.001], alcohol consumption habits [Chi-squared (3) = 800.76, *p*<0.001], age [F (3, 9,059) = 24.99, *p*<0.001], and years of education [F (3, 9,055) = 380.40, *p*<0.001] (Table 1).

**Table 1:** Descriptive statistics.

	China (n = 3,862)		Japan (n = 2,426)		South Korea (n = 1,556)		Taiwan (n = 1,135)		F/Chi^2
	n	%	n	%	n	%	N	%	
Weight status									121.22*
Underweight	432	11.19	223	9.19	112	7.20	65	6.04	
Normal	1,886	48.83	1,239	51.07	775	49.81	441	40.99	
Overweight	711	18.41	493	20.32	314	20.18	218	20.26	
Obese	731	18.93	402	16.57	319	20.50	301	27.97	
Severely obese	102	2.64	69	2.84	36	2.31	51	4.74	
Self-rated health status									1,811.97*
Excellent	954	24.71	70	2.81	335	21.27	34	3.00	
Very good	1,284	33.26	392	15.73	479	30.41	176	15.51	
Good	916	23.72	1,305	52.37	385	24.44	318	28.02	
Fair	547	14.17	627	25.16	232	14.73	443	39.03	
Poor	160	4.14	98	3.93	144	9.14	164	14.45	
Gender: female	1,994	51.58	1,342	53.77	832	52.79	581	51.19	3.67
Married or cohabiting	3,084	80.17	1,805	72.34	1,007	64.10	684	60.37	253.77*
Urban	2,415	62.47	1,619	64.99	1,353	86.29	932	82.84	419.11*
Never smoke	2,658	69.02	1,945	78.05	1,136	72.26	917	80.79	95.38*
Never drink	2,385	62.19	755	30.39	510	32.40	636	50.08	800.76*
	Mean	SD	Mean	SD	Mean	SD	Mean	SD	
Age (years)	47.35	20.65	53.70	16.98	48.94	58.45	47.35	18.17	24.99*
Education (years)	8.58	5.19	13.15	6.68	11.89	4.32	11.28	5.41	380.40*

*p<0.001

Table 2 summarizes the distribution of SRH scores by their weight status stratified by the four countries. The results show that SRH scores were different according to weight status in China [F (df1, df2) = 9.24 (4, 3,852), *p*<0.001], Japan [F (df1, df2) = 4.63 (4, 2,419), *p* = 0.001], and South Korea [F (df1, df2) = 12.22 (4, 1,550), *p*<0.001]. However, this difference was not found in Taiwan [F (df1, df2) = 2.18 (4, 1,071), *p* = 0.070]. Specifically, respondents from China who were underweight reported poorer SRH scores [M (SD) = 3.32 (1.24)] than those who were of normal weight [M (SD) = 3.63 (1.10)], overweight [M (SD) = 3.69 (1.09)], or obese [M (SD) = 3.65 (1.14)]. In Japan, respondents who were obese reported poorer SRH scores [M (SD) = 2.79 (0.72)] than their normal weight counterparts [M (SD) = 2.95 (0.83)], but no differences were found among other weight groups in their SRH scores. Korean respondents with normal weight reported the best SRH scores [M (SD) = 3.56 (1.15)] followed by overweight [M (SD) = 3.47 (1.22)], underweight [M (SD) = 3.24 (1.30)], obese [M (SD) = 3.14 (1.29)], and severely obese [M (SD) = 2.56 (1.21)]. Differences in SRH scores between normal and obese or severely obese groups were significant in South Korea. In Taiwan, however, no group differences in their SRH scores were found among the weight status groups. Furthermore, the best SRH scores were reported by the overweight group in Taiwan or China, whereas the same were found in normal weight groups in Japan or South Korea (Table 2).

**Table 2:** Summary of self-rated health status and weight status by country.

Nation		Total	Weight status					
			Under-weight	Normal weight	Over-weight	Obese	Severely obese	F
China	Mean	3.60	3.32^a,b,c^	3.63^a^	3.69^b^	3.65^c^	3.41	9.24**
	SD	1.13	1.24	1.10	1.09	1.14	1.05	
	n	3,857	431	1,884	711	729	102	
Japan	Mean	2.89	2.90	2.95^a^	2.84	2.79^a^	2.67	4.63*
	SD	0.81	0.90	0.83	0.77	0.72	0.90	
	n	2,424	222	1,239	492	402	69	
South Korea	Mean	3.41	3.24^e^	3.56^a,b^	3.47^c,d^	3.14^a,c^	2.56^b,d,e^	12.22**
	SD	1.22	1.30	1.15	1.22	1.29	1.21	
	n	1,555	112	775	314	318	36	
Taiwan	Mean	2.54	2.43	2.57	2.67	2.47	2.33	2.18
	SD	1.01	0.92	1.00	1.03	1.02	0.95	
	n	1,076	65	441	218	301	51	

Note: Paired groups with the same superscript letters are significantly different (*p*<0.05) with Bonferroni-corrected post-hoc tests

*p<0.01,

**p<0.001

SD, standard deviation

Table 3 presents the results from ordinal regression analyses of SRH scores on their weight status controlling for relevant covariates including age, gender, marital status, regional area, education, and health-related behaviors such as smoking and alcohol consumption. Results showed that, in China, respondents who were underweight [B (SE) = -0.33 (0.10), *p* = 0.001] reported poorer SRH scores while those who were overweight [B (SE) = 0.17 (0.08), *p* = 0.036] reported better scores than those of normal weight after adjusting for other covariates. In Japan, respondents who were obese [B (SE) = -0.24 (0.11), *p* = 0.029] and severely obese [B (SE) = -0.67 (0.25), *p* = 0.006)] reported poorer SRH than their counterparts in the normal weight group. Korean respondents who were underweight [B (SE) = -0.47 (0.19), *p* = 0.013], obese [B (SE) = -0.54 (0.13), *p*<0.001], or severely obese [B (SE) = -1.38 (0.31), *p*<0.001] reported poorer SRH than the normal weight group. In Taiwan, however, no significant differences were found between weight groups compared to the normal weight group (Table 3).

**Table 3:** Ordinal logit regression of self-rated health status on weight status and other covariates by country.

	China (n = 3,807)			Japan (n = 2,405)			South Korea (n = 1,538)			Taiwan (n = 1,061)		
	B	SE (B)	95% CI	B	SE (B)	95% CI	B	SE (B)	95% CI	B	SE (B)	95% CI
Normal: reference												
Underweight	-0.33**	0.10	(-0.53, -0.14)	-0.10	0.14	(-0.38, 0.18)	-0.47*	0.19	(-0.84, -0.1)	-0.27	0.25	(-0.75, 0.22)
Overweight	0.17*	0.08	(0.01, 0.33)	-0.16	0.10	(-0.36, 0.05)	-0.08	0.12	(-0.32, 0.17)	0.13	0.15	(-0.17, 0.43)
Obese	0.14	0.08	(-0.02, 0.3)	-0.24*	0.11	(-0.46, -0.03)	-0.54***	0.13	(-0.79, -0.3)	-0.11	0.14	(-0.39, 0.17)
Severely obese	-0.25	0.18	(-0.61, 0.11)	-0.67**	0.25	(-1.16, -0.19)	-1.38***	0.31	(-1.99, -0.77)	-0.37	0.27	(-0.9, 0.16)
Age	-0.04***	0.00	(-0.04, -0.04)	-0.03***	0.00	(-0.04, -0.03)	0.00**	0.00	(-0.01, 0)	0.00	0.00	(-0.01, 0)
Gender: female	-0.14	0.08	(-0.3, 0.01)	-0.06	0.09	(-0.23, 0.11)	-0.44***	0.12	(-0.67, -0.21)	-0.16	0.13	(-0.41, 0.09)
Married or cohabiting	0.01	0.08	(-0.14, 0.16)	0.18*	0.09	(0.01, 0.36)	-0.21*	0.10	(-0.4, -0.01)	0.28*	0.13	(0.02, 0.53)
Urban	0.08	0.07	(-0.05, 0.22)	0.03	0.08	(-0.14, 0.19)	0.30*	0.15	(0, 0.59)	-0.02	0.16	(-0.33, 0.29)
Education	0.04***	0.01	(0.03, 0.05)	0.01*	0.01	(0, 0.02)	0.16***	0.01	(0.13, 0.18)	0.06***	0.02	(0.03, 0.09)
Never smoke	0.01	0.08	(-0.15, 0.17)	0.20	0.10	(0, 0.39)	0.29*	0.12	(0.05, 0.53)	0.03	0.15	(-0.27, 0.33)
Never drink	-0.38***	0.07	(-0.53, -0.24)	-0.31**	0.09	(-0.49, -0.13)	-0.23*	0.11	(-0.44, -0.01)	-0.19	0.12	(-0.43, 0.05)

*p<0.05,

**p<0.01,

***p<0.001

SE, standard error; CI, confidence interval

## Discussion

The present study was conducted to explore the relationships between BMI and SRH status in four East Asian countries: China, Japan, South Korea, and Taiwan. The findings suggest that the relationships between BMI with SRH status vary across countries.

Traditionally, East Asian cultures, including China, Japan, South Korea, and Taiwan, are strongly influenced by Confucianism. In the view of Confucianism, human beings are “organic and network-based entities that are interconnected with each other, family, community, and society” [16]. However, the Confucian cultural values and politics vary among the four countries based on their Westernization as well as differences in intergeneration gaps. For example, Japan was exposed to Western culture earlier than other countries, and China is the fastest growing country in the world economically [16,17]. East Asian countries are no longer a single cultural entity, but rather unique cultures that share Confucian characteristics [17].

Differences in cultural background and in stages of socioeconomic development may lead to differences in social meanings of weight and interpretations of SRH status [9,16]. The gross domestic product (at purchasing power parity) per capita in China, Japan, South Korea, and Taiwan were $14,450, $40,763, $34,647, and $24,985, respectively, in 2016 [18,19]. Japan is a high-income, developed country, whereas South Korea and Taiwan are high-income developing countries and China is an upper-middle-income developing county [18,20]. People feel pressured by adhering to social norms of being ‘thin’ and stressed by weight-related stigma as the country has advanced [21–23]. Stigma in weight ultimately led to more negative physical and psychological health outcomes [21–23]. Recent studies found that stigma against weight (“anti-fat cultural context [21]”) was extremely high in South Korea [21,24–26] and Japan [27,28], but Chinese were more tolerant about overweight/obesity [9,24,29,30].

In South Korea, a high-income developing country, both underweight and obese/severely obese were significantly associated with poor SRH status in the current study. South Korea is one of the most rapidly aging counties because of decreasing birth rate and increasing life expectancy and has recently experienced rapid growth in its economy [31]. Therefore, the finding regarding the association between being underweight and poor SRH status was consistent with previous findings in low-income or middle-income developing countries [9,32], while the finding regarding the negative association between obese/severely obese and SRH status was consistent with the results of other studies that found that being obese was associated with poor SRH status [5,6,33,34]. In South Korea, especially among the younger generation, people who are more skinny are seen as more attractive [25,26]. Korean showed the highest level of disturbed eating behavior and body dissatisfaction in the world [22]. Young South Koreans are more accepting of cosmetic surgery and, indeed, some view cosmetic surgery as a good way to improve their body image [35]. According to statistics released by the International Society of Aesthetic Plastic Surgery, South Korea has the world’s highest rate of cosmetic plastic surgery [36]. Koreans’ keen interest in appearance may influence their perception of SRH status based on BMI status.

Japan is a very highly developed country, but is aging faster than any other developed country [31]. In the current study, Japanese who were obese/severely obese showed poor SRH status, which was supported by previous studies in USA, Japan, and Singapore, which found a positive relationship between obesity or higher BMI and poor SRH status [6,32,37,38]. Japanese people might perceive they were not healthy when they were obese, which is consistent with the perception that obesity was one of the most serious health problems in developed nations [9].

In contrast to South Korea and Japan, being underweight was a significant factor associated with a poorer SRH after adjusting for covariates in China. This result is consistent with the findings of a previous study that only underweight was associated with poor SRH status [29]. Lee and colleagues analyzed the perception of obesity and body somatotype among university students in China and Korea [24]. Chinese students had a more positive perception towards the overweight somatotype in males than Korean students. Despite China’s rapid economic development, being overweight might still be perceived to be associated with wealth or health in China [9,29]. Chinese people tend to believe that happiness is related to obesity [30]. Chinese respondents who were underweight might have rated their health as poor because they think the wealthy people can afford to buy food or to better maintain their wellness in Chinese culture [24,29,39,40].

Taiwan is a high-income developing country, but there was no significant relationship between obesity and SRH status, unlike South Korea. One study in Taiwan found that being underweight was associated with poor SRH status by both the adolescents and their parents, while overweight and obesity were not associated with poor SRH status [39]; these findings are not consistent with the findings of this study. Little is known about the relationship between obesity and SRH status for adult Taiwanese. Taiwan is undergoing rapid economic growth and is rapidly aging due to increasing life expectancy [31]. Further studies on a representative adult Taiwanese population are needed to explore the relationship between weight status and SRH status.

In a systematic review by Dinsa and colleagues [32], socioeconomic status (SES) was positively associated with obesity in low-income countries, which indicates that overweight or obese might be perceived as a status symbol. But, in the middle-income countries, the association was mixed for men, whereas women with higher SES were less likely to be overweight or obese. Racial/ethnic differences can influence the relationship of obesity with SRH status [14,41,42]. Whites showed negative relationship between SRH status and BMI, whereas Blacks showed positive relationship between SRH status and BMI. There was no significant association between SRH status and BMI in Asians [14]. U-shaped association between BMI and poor SRH status was found in Chinese [43] and in Koreans [44], but, in this study, U-shaped association only found in South Korea. With BMI being an exception, a number of cross-national studies showed the effects of demographic, socioeconomic, and health behaviors on SRH status [11,13].

This study has several limitations. First, this study was a cross-sectional study that could not establish cause-and-effect relationships between BMI and SRH status. Second, this study could not control for other factors that may be related to SRH status, such as medical conditions and physical activity. Lastly, this study measured obesity using self-reported BMI, but did not consider other measures, such as percent body fat or central fat distribution. Thus, BMI might not accurately portray fat mass and, therefore, risk of obesity-related conditions in some individuals, such as those with high amounts of muscle. Despite the limitations, this study was the first to compare the relationship between BMI and SRH status in four East Asian countries using a single data source; previous studies only examined the relationship between BMI and SRH status in one or two countries [24,37,45]. Therefore, the finding that these relationships rely on the unique socioeconomic and cultural context of each nation is meaningful and will guide future studies.

In conclusion, this study revealed that the relationships between BMI and SRH status varied in four neighboring Asian countries that are all influenced by Confucian culture and that interact with each other. Differences in the stage of economic development, the economic growth rate, and health-related culture may influence the differences in the relationship between BMI and SRH status in the four countries. Obesity and SRH status should be interpreted in the economic and cultural context of each country.
